# Crystal structure of bis­[2-(1*H*-benzimid­azol-2-yl)-4-bromo­phenolato-κ^2^ *N* ^3^,*O*]cobalt(II)

**DOI:** 10.1107/S1600536814021813

**Published:** 2014-10-11

**Authors:** Yan Fan, Zhi-Rong Qu

**Affiliations:** a Key Laboratory of Organosilicon Chemistry and Material Technology of the Ministry of Education, Hangzhou Normal University, Hangzhou 311121, People’s Republic of China

**Keywords:** crystal structure, cobalt(II), 2-(1*H*-benzimidazol-2-yl)-4-bromo­phenolate anion, hydrogen bonds, π–π stacking

## Abstract

The asymmetric unit of the title Co^II^ complex, [Co(C_13_H_8_BrN_2_O)_2_], contains two independent mol­ecules (*A* and *B*). In both mol­ecules, the Co^II^ cation is *N*,*O*-chelated by two 2-(1*H*-benzimidazol-2-yl)-4-bromo­phenolate anions in a distorted tetra­hedral geometry. In mol­ecule *A*, both chelating rings display an envelope conformation, with the flap Co atom lying 0.614 (6) and 0.483 (6) Å from the mean planes of the remaining atoms. In mol­ecule *B*, both chelating rings are approximately planar, the maximum deviations being 0.039 (4) and 0.076 (3) Å. In the crystal, mol­ecules are linked by classical N—H⋯O hydrogen bonds and weak C—H⋯O and C—H⋯Br hydrogen bonds into a three-dimensional supra­molecular network. Extensive π–π stacking is observed between nearly parallel aromatic rings of adjacent mol­ecules with centroid–centroid distances in the range 3.407 (3)–3.850 (4) Å.

## Related literature   

For the crystal structures of related metal complexes with the ligand 2-(1*H*-benzoimidazol-2-yl)-4-bromo­phenolate, see: Li *et al.* (2002 ▶); Tong (2007 ▶).
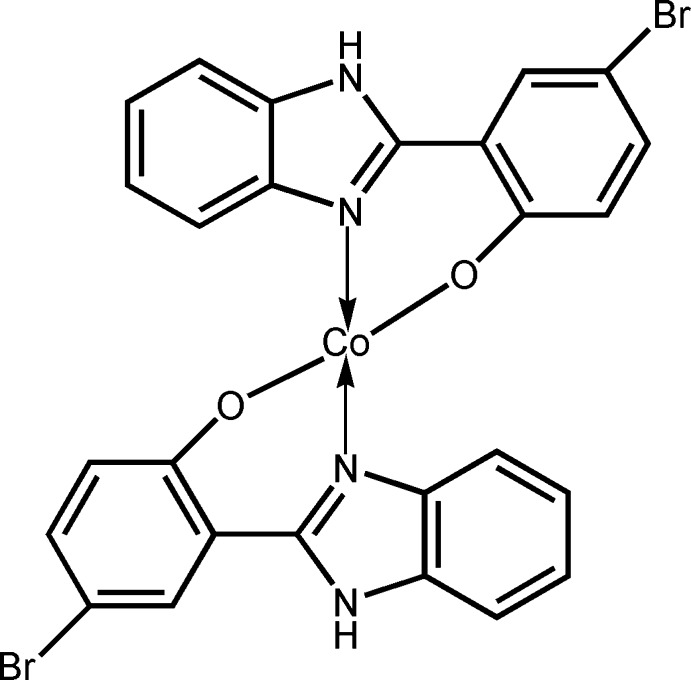


## Experimental   

### Crystal data  

[Co(C_13_H_8_BrN_2_O)_2_]
*M* *_r_* = 635.16
Monoclinic, 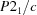

*a* = 22.4334 (16) Å
*b* = 8.3598 (6) Å
*c* = 30.0748 (16) Å
β = 125.323 (4)°
*V* = 4601.9 (5) Å^3^
*Z* = 8
Mo *K*α radiation
μ = 4.25 mm^−1^
*T* = 293 K
0.30 × 0.26 × 0.20 mm

### Data collection  

Bruker APEXII CCD diffractometer
Absorption correction: multi-scan (*SADABS*; Bruker, 2001 ▶) *T*_min_ = 0.258, *T*_max_ = 0.398
29475 measured reflections
10549 independent reflections
5904 reflections with *I* > 2σ(*I*)
*R*_int_ = 0.053

### Refinement  

*R*[*F* ^2^ > 2σ(*F* ^2^)] = 0.046
*wR*(*F* ^2^) = 0.166
*S* = 0.97
10549 reflections
631 parameters
H-atom parameters constrained
Δρ_max_ = 0.71 e Å^−3^
Δρ_min_ = −0.65 e Å^−3^

### 

Data collection: *APEX2* (Bruker, 2007 ▶); cell refinement: *SAINT* (Bruker, 2007 ▶); data reduction: *SAINT*; program(s) used to solve structure: *SHELXTL* (Sheldrick, 2008 ▶); program(s) used to refine structure: *SHELXTL*; molecular graphics: *SHELXTL*; software used to prepare material for publication: *SHELXTL* (Sheldrick, 2008 ▶).

## Supplementary Material

Crystal structure: contains datablock(s) I, New_Global_Publ_Block. DOI: 10.1107/S1600536814021813/xu5818sup1.cif

Structure factors: contains datablock(s) I. DOI: 10.1107/S1600536814021813/xu5818Isup2.hkl

**Fig. 1.**
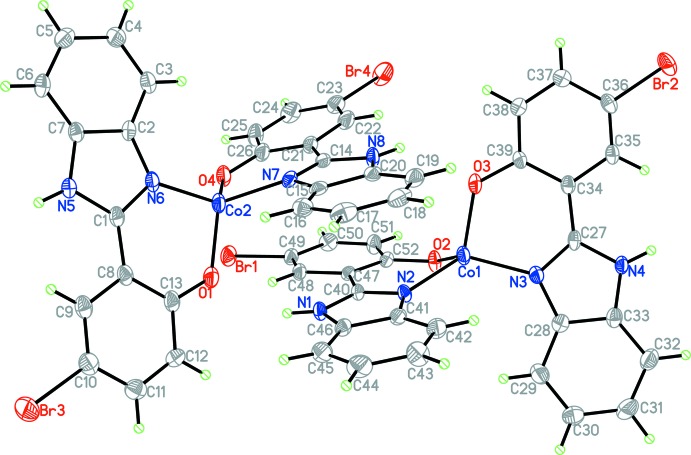
A view of the asymmetric unit of the title compound with atomic numbering scheme. Displacement ellipsoids were drawn at the 30% probability level and all H atoms have been omitted for clarity.

**Fig. 2.**
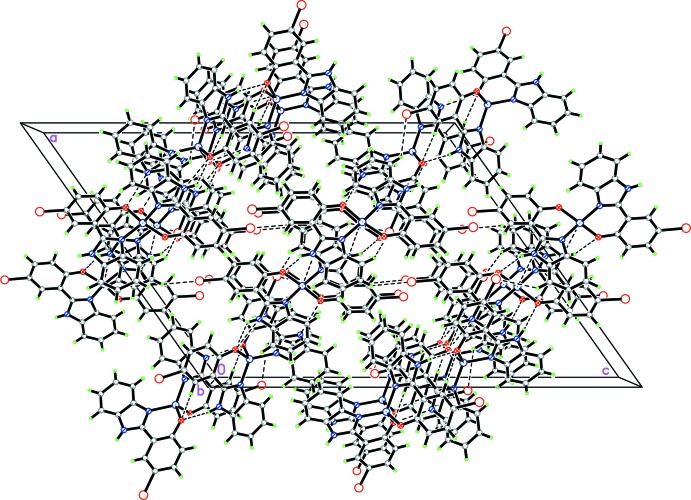
Packing diagram of the title compound viewed along the c axis. Hydrogen bonds are shown as dashed lines.

. DOI: 10.1107/S1600536814021813/xu5818fig1.tif
A view of the asymmetric unit of the title compound with atomic numbering scheme. Displacement ellipsoids were drawn at the 30% probability level and all H atoms have been omitted for clarity.

c . DOI: 10.1107/S1600536814021813/xu5818fig2.tif
Packing diagram of the title compound viewed along the ***c*** axis. Hydrogen bonds are shown as dashed lines.

CCDC reference: 1027432

Additional supporting information:  crystallographic information; 3D view; checkCIF report

## Figures and Tables

**Table 1:** Selected bond lengths ()

Co1O2	1.912(4)
Co1O3	1.903(4)
Co1N2	1.975(4)
Co1N3	1.967(4)
Co2O1	1.930(4)
Co2O4	1.912(4)
Co2N6	1.961(4)
Co2N7	1.957(4)

**Table 2:** Hydrogen-bond geometry (, )

*D*H*A*	*D*H	H*A*	*D* *A*	*D*H*A*
N1H1*A*O1^i^	0.86	2.19	2.895(5)	139
N4H4*A*O2^ii^	0.86	2.09	2.849(5)	147
N5H5*A*O4^iii^	0.86	2.41	3.066(5)	133
N8H8*A*O3^iv^	0.86	2.17	2.808(5)	131
C4H4Br4^i^	0.93	2.88	3.703(6)	149
C6H6Br1^v^	0.93	2.91	3.685(6)	142
C35H35O2^ii^	0.93	2.54	3.394(7)	153

Symmetry codes: (i) 
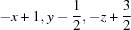
; (ii) 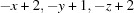; (iii) 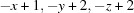; (iv) 
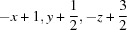
; (v) 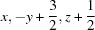.

## supplementary crystallographic information

### S1. Comment

The asymmetric unit of the title compound contains two crystallographically independent molecules and bond lengths and angles are in the normal range (Li *et al.*, 2002; Tong, 2007). The Co(II) atom is coordinated by two N atoms and two O atoms to give a distorted tetrahedral geometry (Fig. 1). The benzimidazole and bromophenyl groups are nearly coplanar; the dihedral angle between them is 4.2 (3)°. The angles between the two ligand planes in each independent molecule are 71.66 (5)° and 63.66 (5)°, respectively. The crystal structure is stabilized by N–H···O hydrogen bonds linking molecules into a three-dimensional network structure (Fig. 2). The structure is further stabilized by π-π stacking interactions, the centroid-to-centroid separations are ranged from 3.407 (3) to 3.850 (4) Å.

### S2. Experimental

Synthesis of the ligand: The ligands were prepared by the reaction of the addition products of 2-hydroxy-5-bromobenzaldehyde (6 mmol 1.2 g) and NaHSO_3_ (6 mmol 0.65 g) were stirred at room temperature in ethanol (25 ml) and a precipitate is formed after 4 h reaction, then, *o*-phenylenediamine (6 mmol, 0.65 g) and 25 ml DMF were added to this mixture. After 2 h reflux the solution was poured into 10-times water. The benzimidazole compound was filtered, dried and crystallized from ethanol.

Synthesis of the complex [Co(C_26_H_16_N_4_O_2_Br_2_)]_2_: 2-(1*H*-benzimidazol-2-yl)-4-bromophenol (0.2 mmol, 58 mg) was dissolved in DMF (6 ml) and CoCl_2_.6H_2_O (0.1 mmol 24 mg) was dissolved in H_2_O (6 ml), and the mixture poured into a 25 ml dicting kettle, then maintaining 393 K for 3 d, allowed to reach room temperate, and red block-shaped crystals suitable for X-ray diffraction were obtained.

### S3. Refinement

H atoms were placed in calculated positions with C—H = 0.97-0.93 Å and N—H = 0.86 Å, and refined in riding mode, U_iso_(H) = 1.2U_eq_(C,N).

### Crystal data

**Table:**

[Co(C_13_H_8_BrN_2_O)_2_]	*F*(000) = 2503
*M**_r_* = 635.16	*D*_x_ = 1.834 Mg m^−^^3^
Monoclinic, *P*2_1_/*c*	Mo *K*α radiation, λ = 0.71073 Å
*a* = 22.4334 (16) Å	Cell parameters from 29475 reflections
*b* = 8.3598 (6) Å	θ = 1.1–27.5°
*c* = 30.0748 (16) Å	µ = 4.25 mm^−^^1^
β = 125.323 (4)°	*T* = 293 K
*V* = 4601.9 (5) Å^3^	Block, red
*Z* = 8	0.30 × 0.26 × 0.20 mm

### Data collection

**Table:**

Bruker APEXII CCD diffractometer	10549 independent reflections
Radiation source: fine-focus sealed tube	5904 reflections with *I* > 2σ(*I*)
Graphite monochromator	*R*_int_ = 0.053
φ and ω scans	θ_max_ = 27.5°, θ_min_ = 1.1°
Absorption correction: multi-scan (*SADABS*; Bruker, 2001)	*h* = −29→22
*T*_min_ = 0.258, *T*_max_ = 0.398	*k* = −10→10
29475 measured reflections	*l* = −36→39

### Refinement

**Table:**

Refinement on *F*^2^	Primary atom site location: structure-invariant direct methods
Least-squares matrix: full	Secondary atom site location: difference Fourier map
*R*[*F*^2^ > 2σ(*F*^2^)] = 0.046	Hydrogen site location: inferred from neighbouring sites
*wR*(*F*^2^) = 0.166	H-atom parameters constrained
*S* = 0.97	*w* = 1/[σ^2^(*F*_o_^2^) + (0.0908*P*)^2^] where *P* = (*F*_o_^2^ + 2*F*_c_^2^)/3
10549 reflections	(Δ/σ)_max_ = 0.001
631 parameters	Δρ_max_ = 0.71 e Å^−^^3^
0 restraints	Δρ_min_ = −0.65 e Å^−^^3^

### Special details

**Table:**

Geometry. All e.s.d.'s (except the e.s.d. in the dihedral angle between two l.s. planes) are estimated using the full covariance matrix. The cell e.s.d.'s are taken into account individually in the estimation of e.s.d.'s in distances, angles and torsion angles; correlations between e.s.d.'s in cell parameters are only used when they are defined by crystal symmetry. An approximate (isotropic) treatment of cell e.s.d.'s is used for estimating e.s.d.'s involving l.s. planes.
Refinement. Refinement of *F*^2^ against ALL reflections. The weighted *R*-factor *wR* and goodness of fit *S* are based on *F*^2^, conventional *R*-factors *R* are based on *F*, with *F* set to zero for negative *F*^2^. The threshold expression of *F*^2^ > σ(*F*^2^) is used only for calculating *R*-factors(gt) *etc*. and is not relevant to the choice of reflections for refinement. *R*-factors based on *F*^2^ are statistically about twice as large as those based on *F*, and *R*- factors based on ALL data will be even larger.

### Fractional atomic coordinates and isotropic or equivalent isotropic displacement parameters (Å^2^)

**Table:**

	*x*	*y*	*z*	*U*_iso_*/*U*_eq_	
Br1	0.58763 (3)	0.94519 (8)	0.67243 (2)	0.04708 (19)	
Br2	0.96705 (4)	−0.01562 (9)	1.09681 (2)	0.0546 (2)	
Co1	0.90832 (4)	0.46833 (10)	0.87113 (3)	0.0345 (2)	
O2	0.85783 (19)	0.6568 (5)	0.86818 (12)	0.0383 (10)	
O3	0.86633 (19)	0.2923 (5)	0.88397 (12)	0.0439 (10)	
N2	0.8868 (2)	0.5101 (5)	0.79854 (15)	0.0278 (10)	
N1	0.8152 (2)	0.5734 (5)	0.71102 (15)	0.0298 (10)	
H1A	0.7795	0.6141	0.6809	0.036*	
N3	1.0079 (2)	0.4220 (5)	0.93502 (15)	0.0321 (10)	
N4	1.0909 (2)	0.3509 (5)	1.02030 (15)	0.0312 (10)	
H4A	1.1100	0.3093	1.0520	0.037*	
C41	0.9158 (3)	0.4410 (7)	0.77260 (19)	0.0354 (13)	
C28	1.0753 (3)	0.4820 (7)	0.9510 (2)	0.0337 (13)	
C36	0.9396 (3)	0.0799 (7)	1.0302 (2)	0.0380 (14)	
C40	0.8271 (3)	0.5913 (6)	0.76042 (17)	0.0260 (11)	
C27	1.0192 (3)	0.3386 (7)	0.97719 (17)	0.0298 (12)	
C46	0.8705 (3)	0.4784 (7)	0.7173 (2)	0.0301 (12)	
C45	0.8855 (3)	0.4264 (8)	0.6813 (2)	0.0437 (15)	
H45	0.8550	0.4512	0.6444	0.052*	
C48	0.7168 (3)	0.7559 (7)	0.72527 (18)	0.0340 (13)	
H48	0.7050	0.7419	0.6904	0.041*	
C33	1.1276 (3)	0.4405 (7)	1.0050 (2)	0.0365 (13)	
C49	0.6728 (3)	0.8449 (7)	0.73248 (19)	0.0335 (13)	
C35	0.9864 (3)	0.1691 (7)	1.02718 (18)	0.0315 (12)	
H35	1.0341	0.1807	1.0580	0.038*	
C34	0.9655 (3)	0.2465 (7)	0.97832 (17)	0.0303 (12)	
C43	0.9927 (3)	0.2955 (8)	0.7576 (2)	0.0488 (16)	
H43	1.0336	0.2323	0.7702	0.059*	
C44	0.9470 (3)	0.3370 (9)	0.7022 (2)	0.0534 (18)	
H44	0.9591	0.3023	0.6790	0.064*	
C31	1.2178 (3)	0.5794 (9)	1.0040 (3)	0.0572 (18)	
H31	1.2655	0.6161	1.0215	0.069*	
C50	0.6885 (3)	0.8666 (7)	0.7841 (2)	0.0408 (14)	
H50	0.6569	0.9242	0.7886	0.049*	
C47	0.7801 (2)	0.6842 (6)	0.76986 (17)	0.0254 (11)	
C42	0.9773 (3)	0.3479 (8)	0.7936 (2)	0.0468 (16)	
H42	1.0071	0.3213	0.8304	0.056*	
C37	0.8683 (3)	0.0559 (7)	0.9848 (2)	0.0454 (15)	
H37	0.8360	−0.0078	0.9869	0.054*	
C51	0.7506 (3)	0.8027 (7)	0.82781 (19)	0.0388 (14)	
H51	0.7617	0.8204	0.8624	0.047*	
C32	1.2000 (3)	0.4847 (8)	1.0325 (3)	0.0505 (17)	
H32	1.2349	0.4526	1.0682	0.061*	
C39	0.8932 (3)	0.2255 (7)	0.93201 (18)	0.0326 (13)	
C52	0.7979 (3)	0.7121 (6)	0.82274 (18)	0.0292 (12)	
C29	1.0957 (3)	0.5776 (8)	0.9235 (2)	0.0474 (16)	
H29	1.0613	0.6101	0.8877	0.057*	
C30	1.1663 (3)	0.6213 (10)	0.9498 (3)	0.063 (2)	
H30	1.1808	0.6805	0.9314	0.076*	
C38	0.8472 (3)	0.1294 (8)	0.9369 (2)	0.0451 (15)	
H38	0.7997	0.1137	0.9063	0.054*	
Br3	0.33496 (4)	1.05417 (12)	1.10266 (3)	0.0769 (3)	
Br4	0.39831 (4)	0.96643 (12)	0.64170 (3)	0.0711 (3)	
Co2	0.37925 (4)	0.92802 (10)	0.87904 (3)	0.0363 (2)	
O1	0.3195 (2)	1.0374 (5)	0.89651 (14)	0.0451 (11)	
O4	0.4319 (2)	1.0277 (5)	0.85430 (14)	0.0430 (10)	
N6	0.4425 (2)	0.8299 (6)	0.95131 (15)	0.0340 (11)	
N5	0.4949 (2)	0.7840 (5)	1.03884 (15)	0.0323 (10)	
H5A	0.5032	0.7876	1.0705	0.039*	
N7	0.3160 (2)	0.8034 (6)	0.81237 (15)	0.0339 (11)	
N8	0.2534 (2)	0.7465 (6)	0.72462 (15)	0.0356 (11)	
H8A	0.2400	0.7475	0.6914	0.043*	
C13	0.3259 (3)	1.0325 (7)	0.9430 (2)	0.0382 (14)	
C21	0.3638 (3)	0.9139 (7)	0.76286 (19)	0.0359 (13)	
C14	0.3128 (3)	0.8257 (7)	0.76724 (18)	0.0324 (13)	
C8	0.3808 (3)	0.9497 (7)	0.9903 (2)	0.0355 (13)	
C3	0.5332 (3)	0.6743 (7)	0.9445 (2)	0.0425 (15)	
H3	0.5116	0.6953	0.9078	0.051*	
C19	0.1575 (3)	0.5672 (7)	0.7178 (2)	0.0435 (15)	
H19	0.1312	0.5432	0.6809	0.052*	
C6	0.5994 (3)	0.6084 (7)	1.0561 (2)	0.0409 (15)	
H6	0.6213	0.5861	1.0928	0.049*	
C2	0.5037 (3)	0.7354 (7)	0.97079 (18)	0.0314 (12)	
C9	0.3807 (3)	0.9555 (7)	1.0370 (2)	0.0393 (14)	
H9	0.4166	0.9005	1.0682	0.047*	
C4	0.5948 (3)	0.5823 (8)	0.9741 (2)	0.0478 (16)	
H4	0.6156	0.5406	0.9573	0.057*	
C15	0.2582 (3)	0.7042 (7)	0.79894 (19)	0.0344 (13)	
C26	0.4212 (3)	1.0058 (8)	0.8068 (2)	0.0404 (15)	
C1	0.4374 (3)	0.8591 (7)	0.99257 (18)	0.0319 (12)	
C10	0.3293 (3)	1.0395 (8)	1.0377 (2)	0.0455 (16)	
C17	0.1765 (3)	0.5397 (7)	0.8052 (3)	0.0471 (16)	
H17	0.1611	0.4947	0.8253	0.057*	
C20	0.2184 (3)	0.6648 (6)	0.7434 (2)	0.0303 (12)	
C24	0.4628 (3)	1.0748 (9)	0.7502 (3)	0.0521 (17)	
H24	0.4951	1.1293	0.7457	0.062*	
C5	0.6266 (3)	0.5506 (8)	1.0284 (2)	0.0478 (16)	
H5	0.6684	0.4873	1.0471	0.057*	
C7	0.5373 (3)	0.7018 (7)	1.02606 (18)	0.0307 (12)	
C25	0.4695 (3)	1.0851 (8)	0.7981 (2)	0.0454 (16)	
H25	0.5072	1.1465	0.8261	0.054*	
C16	0.2367 (3)	0.6371 (7)	0.8302 (2)	0.0381 (13)	
H16	0.2628	0.6582	0.8673	0.046*	
C22	0.3593 (3)	0.9059 (9)	0.7142 (2)	0.0464 (16)	
H22	0.3219	0.8463	0.6853	0.056*	
C12	0.2744 (3)	1.1173 (8)	0.9458 (2)	0.0425 (14)	
H12	0.2374	1.1715	0.9150	0.051*	
C23	0.4072 (3)	0.9819 (8)	0.7081 (2)	0.0457 (16)	
C11	0.2759 (3)	1.1239 (8)	0.9918 (2)	0.0445 (15)	
H11	0.2416	1.1844	0.9925	0.053*	
C18	0.1387 (3)	0.5089 (8)	0.7500 (3)	0.0480 (16)	
H18	0.0976	0.4437	0.7340	0.058*	

### Atomic displacement parameters (Å^2^)

**Table:**

	*U*^11^	*U*^22^	*U*^33^	*U*^12^	*U*^13^	*U*^23^
Br1	0.0315 (3)	0.0561 (5)	0.0418 (3)	0.0132 (3)	0.0143 (3)	0.0101 (3)
Br2	0.0679 (5)	0.0629 (5)	0.0378 (3)	0.0107 (4)	0.0334 (3)	0.0182 (3)
Co1	0.0303 (4)	0.0474 (5)	0.0209 (3)	0.0058 (4)	0.0121 (3)	0.0074 (3)
O2	0.036 (2)	0.052 (3)	0.0194 (16)	0.0137 (19)	0.0118 (15)	0.0036 (16)
O3	0.034 (2)	0.063 (3)	0.0200 (16)	−0.001 (2)	0.0066 (15)	0.0088 (17)
N2	0.027 (2)	0.031 (3)	0.0247 (19)	0.010 (2)	0.0150 (18)	0.0085 (18)
N1	0.025 (2)	0.039 (3)	0.0226 (19)	0.010 (2)	0.0126 (17)	0.0071 (18)
N3	0.032 (2)	0.038 (3)	0.0239 (19)	0.002 (2)	0.0144 (18)	0.0046 (19)
N4	0.026 (2)	0.039 (3)	0.0194 (18)	0.002 (2)	0.0085 (18)	0.0029 (18)
C41	0.029 (3)	0.055 (4)	0.026 (2)	−0.006 (3)	0.017 (2)	−0.002 (2)
C28	0.033 (3)	0.036 (3)	0.033 (3)	0.002 (3)	0.019 (2)	−0.002 (2)
C36	0.047 (3)	0.042 (4)	0.027 (2)	0.005 (3)	0.023 (3)	0.006 (2)
C40	0.024 (3)	0.027 (3)	0.022 (2)	0.005 (2)	0.010 (2)	0.004 (2)
C27	0.029 (3)	0.035 (3)	0.017 (2)	0.008 (2)	0.009 (2)	−0.002 (2)
C46	0.023 (3)	0.031 (3)	0.034 (3)	0.005 (2)	0.015 (2)	0.002 (2)
C45	0.045 (3)	0.061 (5)	0.035 (3)	−0.005 (3)	0.029 (3)	−0.007 (3)
C48	0.028 (3)	0.049 (4)	0.022 (2)	−0.002 (3)	0.012 (2)	0.001 (2)
C33	0.031 (3)	0.042 (4)	0.032 (3)	0.009 (3)	0.016 (2)	0.003 (2)
C49	0.024 (3)	0.043 (4)	0.030 (2)	0.003 (3)	0.013 (2)	0.002 (2)
C35	0.030 (3)	0.041 (4)	0.021 (2)	0.010 (3)	0.013 (2)	0.002 (2)
C34	0.030 (3)	0.037 (3)	0.021 (2)	0.004 (2)	0.013 (2)	0.000 (2)
C43	0.047 (4)	0.050 (4)	0.060 (4)	0.023 (3)	0.036 (3)	0.011 (3)
C44	0.053 (4)	0.072 (5)	0.055 (4)	−0.002 (4)	0.043 (3)	−0.010 (3)
C31	0.034 (3)	0.068 (5)	0.067 (4)	0.003 (3)	0.028 (3)	0.003 (4)
C50	0.042 (3)	0.043 (4)	0.044 (3)	0.001 (3)	0.029 (3)	−0.007 (3)
C47	0.022 (2)	0.029 (3)	0.024 (2)	−0.003 (2)	0.013 (2)	−0.003 (2)
C42	0.033 (3)	0.071 (5)	0.043 (3)	0.006 (3)	0.026 (3)	0.008 (3)
C37	0.041 (3)	0.049 (4)	0.041 (3)	−0.006 (3)	0.021 (3)	0.007 (3)
C51	0.038 (3)	0.055 (4)	0.024 (2)	0.005 (3)	0.018 (2)	−0.004 (2)
C32	0.031 (3)	0.069 (5)	0.045 (3)	0.005 (3)	0.018 (3)	0.004 (3)
C39	0.033 (3)	0.039 (4)	0.022 (2)	0.002 (3)	0.013 (2)	0.005 (2)
C52	0.024 (3)	0.031 (3)	0.026 (2)	−0.001 (2)	0.011 (2)	−0.002 (2)
C29	0.049 (4)	0.048 (4)	0.045 (3)	0.006 (3)	0.027 (3)	0.012 (3)
C30	0.041 (4)	0.097 (6)	0.063 (4)	0.004 (4)	0.037 (3)	0.020 (4)
C38	0.033 (3)	0.054 (4)	0.035 (3)	−0.008 (3)	0.011 (3)	0.006 (3)
Br3	0.0629 (5)	0.1340 (9)	0.0506 (4)	0.0121 (5)	0.0425 (4)	−0.0018 (4)
Br4	0.0482 (4)	0.1339 (8)	0.0419 (3)	0.0164 (4)	0.0321 (3)	0.0144 (4)
Co2	0.0329 (4)	0.0487 (5)	0.0187 (3)	−0.0001 (4)	0.0099 (3)	−0.0015 (3)
O1	0.036 (2)	0.066 (3)	0.0241 (17)	0.003 (2)	0.0122 (16)	−0.0008 (18)
O4	0.035 (2)	0.061 (3)	0.0247 (17)	−0.005 (2)	0.0129 (16)	−0.0056 (18)
N6	0.030 (2)	0.047 (3)	0.0204 (19)	−0.006 (2)	0.0116 (18)	−0.0050 (19)
N5	0.031 (2)	0.038 (3)	0.0220 (19)	−0.002 (2)	0.0120 (18)	−0.0006 (18)
N7	0.027 (2)	0.046 (3)	0.0221 (19)	0.012 (2)	0.0103 (18)	0.0069 (19)
N8	0.029 (2)	0.054 (3)	0.0173 (18)	−0.005 (2)	0.0098 (18)	−0.004 (2)
C13	0.030 (3)	0.049 (4)	0.029 (3)	−0.012 (3)	0.014 (2)	−0.010 (2)
C21	0.027 (3)	0.053 (4)	0.025 (2)	0.005 (3)	0.014 (2)	0.001 (2)
C14	0.029 (3)	0.046 (4)	0.022 (2)	0.013 (3)	0.014 (2)	0.003 (2)
C8	0.027 (3)	0.047 (4)	0.029 (2)	−0.014 (3)	0.014 (2)	−0.011 (2)
C3	0.044 (3)	0.051 (4)	0.027 (3)	−0.003 (3)	0.017 (3)	−0.005 (3)
C19	0.032 (3)	0.035 (4)	0.050 (3)	0.001 (3)	0.016 (3)	−0.007 (3)
C6	0.041 (3)	0.050 (4)	0.024 (2)	−0.001 (3)	0.014 (2)	0.007 (2)
C2	0.028 (3)	0.035 (3)	0.022 (2)	−0.005 (3)	0.009 (2)	0.000 (2)
C9	0.037 (3)	0.047 (4)	0.031 (3)	−0.013 (3)	0.019 (2)	−0.006 (2)
C4	0.045 (4)	0.064 (5)	0.038 (3)	0.007 (3)	0.026 (3)	−0.003 (3)
C15	0.034 (3)	0.036 (4)	0.031 (3)	0.019 (3)	0.018 (2)	0.010 (2)
C26	0.031 (3)	0.061 (4)	0.025 (2)	0.006 (3)	0.014 (2)	0.002 (3)
C1	0.028 (3)	0.038 (4)	0.025 (2)	−0.010 (3)	0.012 (2)	−0.006 (2)
C10	0.036 (3)	0.061 (5)	0.042 (3)	−0.013 (3)	0.024 (3)	−0.010 (3)
C17	0.047 (4)	0.031 (4)	0.072 (4)	0.005 (3)	0.039 (3)	0.012 (3)
C20	0.027 (3)	0.021 (3)	0.038 (3)	0.001 (2)	0.016 (2)	−0.003 (2)
C24	0.037 (3)	0.072 (5)	0.057 (4)	−0.003 (3)	0.032 (3)	0.009 (3)
C5	0.037 (3)	0.055 (5)	0.044 (3)	0.009 (3)	0.020 (3)	0.005 (3)
C7	0.028 (3)	0.036 (3)	0.026 (2)	−0.009 (3)	0.015 (2)	−0.004 (2)
C25	0.032 (3)	0.058 (4)	0.038 (3)	−0.017 (3)	0.015 (3)	−0.005 (3)
C16	0.042 (3)	0.031 (3)	0.040 (3)	0.006 (3)	0.023 (3)	0.006 (3)
C22	0.035 (3)	0.073 (5)	0.029 (3)	0.012 (3)	0.018 (3)	0.007 (3)
C12	0.034 (3)	0.044 (4)	0.041 (3)	0.009 (3)	0.017 (3)	0.002 (3)
C23	0.038 (3)	0.068 (5)	0.038 (3)	0.009 (3)	0.026 (3)	0.008 (3)
C11	0.039 (3)	0.047 (4)	0.051 (3)	−0.005 (3)	0.028 (3)	−0.005 (3)
C18	0.032 (3)	0.038 (4)	0.068 (4)	0.004 (3)	0.025 (3)	0.000 (3)

### Geometric parameters (Å, º)

**Table:**

Br1—C49	1.904 (5)	Br3—C10	1.889 (6)
Br2—C36	1.898 (5)	Br4—C23	1.893 (6)
Co1—O2	1.912 (4)	Co2—O1	1.930 (4)
Co1—O3	1.903 (4)	Co2—O4	1.912 (4)
Co1—N2	1.975 (4)	Co2—N6	1.961 (4)
Co1—N3	1.967 (4)	Co2—N7	1.957 (4)
O2—C52	1.328 (5)	O1—C13	1.322 (6)
O3—C39	1.325 (5)	O4—C26	1.316 (6)
N2—C40	1.339 (6)	N6—C1	1.332 (6)
N2—C41	1.399 (6)	N6—C2	1.388 (7)
N1—C40	1.358 (6)	N5—C1	1.385 (6)
N1—C46	1.390 (6)	N5—C7	1.396 (7)
N1—H1A	0.8600	N5—H5A	0.8600
N3—C27	1.336 (6)	N7—C14	1.330 (6)
N3—C28	1.389 (7)	N7—C15	1.388 (7)
N4—C27	1.366 (6)	N8—C14	1.372 (6)
N4—C33	1.375 (7)	N8—C20	1.384 (7)
N4—H4A	0.8600	N8—H8A	0.8600
C41—C42	1.378 (8)	C13—C12	1.400 (8)
C41—C46	1.392 (7)	C13—C8	1.412 (8)
C28—C33	1.388 (7)	C21—C22	1.408 (7)
C28—C29	1.406 (8)	C21—C26	1.424 (8)
C36—C35	1.334 (8)	C21—C14	1.430 (8)
C36—C37	1.391 (8)	C8—C9	1.406 (7)
C40—C47	1.462 (7)	C8—C1	1.447 (8)
C27—C34	1.448 (7)	C3—C4	1.369 (8)
C46—C45	1.377 (7)	C3—C2	1.389 (7)
C45—C44	1.362 (8)	C3—H3	0.9300
C45—H45	0.9300	C19—C18	1.351 (9)
C48—C49	1.350 (7)	C19—C20	1.382 (8)
C48—C47	1.405 (7)	C19—H19	0.9300
C48—H48	0.9300	C6—C5	1.376 (8)
C33—C32	1.381 (8)	C6—C7	1.381 (7)
C49—C50	1.391 (7)	C6—H6	0.9300
C35—C34	1.414 (6)	C2—C7	1.401 (6)
C35—H35	0.9300	C9—C10	1.359 (8)
C34—C39	1.410 (7)	C9—H9	0.9300
C43—C42	1.385 (8)	C4—C5	1.378 (8)
C43—C44	1.403 (8)	C4—H4	0.9300
C43—H43	0.9300	C15—C16	1.399 (7)
C44—H44	0.9300	C15—C20	1.403 (7)
C31—C32	1.386 (9)	C26—C25	1.420 (8)
C31—C30	1.391 (9)	C10—C11	1.387 (8)
C31—H31	0.9300	C17—C16	1.370 (8)
C50—C51	1.354 (7)	C17—C18	1.385 (9)
C50—H50	0.9300	C17—H17	0.9300
C47—C52	1.417 (6)	C24—C25	1.361 (8)
C42—H42	0.9300	C24—C23	1.391 (9)
C37—C38	1.375 (7)	C24—H24	0.9300
C37—H37	0.9300	C5—H5	0.9300
C51—C52	1.383 (7)	C25—H25	0.9300
C51—H51	0.9300	C16—H16	0.9300
C32—H32	0.9300	C22—C23	1.351 (9)
C39—C38	1.382 (8)	C22—H22	0.9300
C29—C30	1.347 (8)	C12—C11	1.364 (8)
C29—H29	0.9300	C12—H12	0.9300
C30—H30	0.9300	C11—H11	0.9300
C38—H38	0.9300	C18—H18	0.9300
			
O3—Co1—O2	107.28 (18)	O4—Co2—O1	125.57 (19)
O3—Co1—N3	93.74 (17)	O4—Co2—N7	94.49 (17)
O2—Co1—N3	121.01 (17)	O1—Co2—N7	108.97 (17)
O3—Co1—N2	121.83 (17)	O4—Co2—N6	113.60 (17)
O2—Co1—N2	92.47 (15)	O1—Co2—N6	93.86 (17)
N3—Co1—N2	121.93 (17)	N7—Co2—N6	123.0 (2)
C52—O2—Co1	123.7 (3)	C13—O1—Co2	127.3 (4)
C39—O3—Co1	125.5 (3)	C26—O4—Co2	126.5 (4)
C40—N2—C41	106.9 (4)	C1—N6—C2	107.9 (4)
C40—N2—Co1	121.4 (3)	C1—N6—Co2	124.0 (4)
C41—N2—Co1	130.4 (3)	C2—N6—Co2	127.7 (3)
C40—N1—C46	108.6 (4)	C1—N5—C7	109.5 (4)
C40—N1—H1A	125.7	C1—N5—H5A	125.3
C46—N1—H1A	125.7	C7—N5—H5A	125.3
C27—N3—C28	106.7 (4)	C14—N7—C15	107.2 (4)
C27—N3—Co1	121.0 (4)	C14—N7—Co2	123.4 (4)
C28—N3—Co1	131.4 (3)	C15—N7—Co2	128.2 (3)
C27—N4—C33	109.1 (4)	C14—N8—C20	109.3 (4)
C27—N4—H4A	125.4	C14—N8—H8A	125.4
C33—N4—H4A	125.4	C20—N8—H8A	125.4
C42—C41—C46	121.4 (5)	O1—C13—C12	117.4 (5)
C42—C41—N2	130.0 (5)	O1—C13—C8	125.4 (5)
C46—C41—N2	108.6 (5)	C12—C13—C8	117.2 (5)
C33—C28—N3	109.2 (5)	C22—C21—C26	118.3 (5)
C33—C28—C29	119.2 (5)	C22—C21—C14	119.4 (5)
N3—C28—C29	131.5 (5)	C26—C21—C14	122.3 (5)
C35—C36—C37	121.1 (5)	N7—C14—N8	109.7 (5)
C35—C36—Br2	121.3 (4)	N7—C14—C21	126.3 (5)
C37—C36—Br2	117.6 (4)	N8—C14—C21	124.0 (4)
N2—C40—N1	110.3 (4)	C9—C8—C13	119.0 (5)
N2—C40—C47	125.4 (4)	C9—C8—C1	118.7 (5)
N1—C40—C47	124.3 (4)	C13—C8—C1	122.3 (5)
N3—C27—N4	109.6 (5)	C4—C3—C2	118.1 (5)
N3—C27—C34	126.6 (4)	C4—C3—H3	121.0
N4—C27—C34	123.9 (4)	C2—C3—H3	121.0
C45—C46—N1	132.6 (5)	C18—C19—C20	115.6 (6)
C45—C46—C41	121.7 (5)	C18—C19—H19	122.2
N1—C46—C41	105.7 (4)	C20—C19—H19	122.2
C44—C45—C46	116.9 (5)	C5—C6—C7	115.8 (5)
C44—C45—H45	121.5	C5—C6—H6	122.1
C46—C45—H45	121.5	C7—C6—H6	122.1
C49—C48—C47	120.8 (4)	N6—C2—C3	130.6 (4)
C49—C48—H48	119.6	N6—C2—C7	109.7 (4)
C47—C48—H48	119.6	C3—C2—C7	119.7 (5)
N4—C33—C32	132.0 (5)	C10—C9—C8	121.5 (6)
N4—C33—C28	105.2 (5)	C10—C9—H9	119.2
C32—C33—C28	122.7 (6)	C8—C9—H9	119.2
C48—C49—C50	120.9 (5)	C3—C4—C5	121.0 (6)
C48—C49—Br1	121.1 (4)	C3—C4—H4	119.5
C50—C49—Br1	118.0 (4)	C5—C4—H4	119.5
C36—C35—C34	121.7 (5)	N7—C15—C16	132.2 (5)
C36—C35—H35	119.2	N7—C15—C20	109.4 (5)
C34—C35—H35	119.2	C16—C15—C20	118.4 (5)
C39—C34—C35	118.1 (5)	O4—C26—C25	117.2 (5)
C39—C34—C27	122.7 (4)	O4—C26—C21	125.6 (5)
C35—C34—C27	119.1 (4)	C25—C26—C21	117.2 (5)
C42—C43—C44	120.6 (6)	N6—C1—N5	108.8 (5)
C42—C43—H43	119.7	N6—C1—C8	126.7 (5)
C44—C43—H43	119.7	N5—C1—C8	124.5 (4)
C45—C44—C43	122.3 (5)	C9—C10—C11	120.1 (5)
C45—C44—H44	118.9	C9—C10—Br3	119.9 (5)
C43—C44—H44	118.9	C11—C10—Br3	119.8 (5)
C32—C31—C30	121.9 (6)	C16—C17—C18	119.6 (6)
C32—C31—H31	119.1	C16—C17—H17	120.2
C30—C31—H31	119.1	C18—C17—H17	120.2
C51—C50—C49	119.2 (5)	C19—C20—N8	132.4 (5)
C51—C50—H50	120.4	C19—C20—C15	123.1 (5)
C49—C50—H50	120.4	N8—C20—C15	104.5 (4)
C48—C47—C52	118.3 (4)	C25—C24—C23	119.4 (6)
C48—C47—C40	119.1 (4)	C25—C24—H24	120.3
C52—C47—C40	122.5 (4)	C23—C24—H24	120.3
C41—C42—C43	117.1 (5)	C6—C5—C4	123.0 (6)
C41—C42—H42	121.5	C6—C5—H5	118.5
C43—C42—H42	121.5	C4—C5—H5	118.5
C38—C37—C36	118.0 (6)	C6—C7—N5	133.4 (4)
C38—C37—H37	121.0	C6—C7—C2	122.5 (5)
C36—C37—H37	121.0	N5—C7—C2	104.1 (4)
C50—C51—C52	122.2 (5)	C24—C25—C26	122.5 (6)
C50—C51—H51	118.9	C24—C25—H25	118.7
C52—C51—H51	118.9	C26—C25—H25	118.7
C33—C32—C31	116.2 (6)	C17—C16—C15	119.0 (5)
C33—C32—H32	121.9	C17—C16—H16	120.5
C31—C32—H32	121.9	C15—C16—H16	120.5
O3—C39—C38	117.6 (5)	C23—C22—C21	122.3 (6)
O3—C39—C34	124.3 (5)	C23—C22—H22	118.8
C38—C39—C34	118.1 (4)	C21—C22—H22	118.8
O2—C52—C51	117.5 (4)	C11—C12—C13	123.0 (5)
O2—C52—C47	124.0 (5)	C11—C12—H12	118.5
C51—C52—C47	118.5 (4)	C13—C12—H12	118.5
C30—C29—C28	118.7 (6)	C22—C23—C24	120.3 (6)
C30—C29—H29	120.7	C22—C23—Br4	120.3 (5)
C28—C29—H29	120.7	C24—C23—Br4	119.4 (5)
C29—C30—C31	121.2 (6)	C12—C11—C10	119.1 (6)
C29—C30—H30	119.4	C12—C11—H11	120.4
C31—C30—H30	119.4	C10—C11—H11	120.4
C37—C38—C39	123.0 (5)	C19—C18—C17	124.3 (6)
C37—C38—H38	118.5	C19—C18—H18	117.8
C39—C38—H38	118.5	C17—C18—H18	117.8

### Hydrogen-bond geometry (Å, º)

**Table:**

*D*—H···*A*	*D*—H	H···*A*	*D*···*A*	*D*—H···*A*
N1—H1*A*···O1^i^	0.86	2.19	2.895 (5)	139
N4—H4*A*···O2^ii^	0.86	2.09	2.849 (5)	147
N5—H5*A*···O4^iii^	0.86	2.41	3.066 (5)	133
N8—H8*A*···O3^iv^	0.86	2.17	2.808 (5)	131
C4—H4···Br4^i^	0.93	2.88	3.703 (6)	149
C6—H6···Br1^v^	0.93	2.91	3.685 (6)	142
C35—H35···O2^ii^	0.93	2.54	3.394 (7)	153

Symmetry codes: (i) −*x*+1, *y*−1/2, −*z*+3/2; (ii) −*x*+2, −*y*+1, −*z*+2; (iii) −*x*+1, −*y*+2, −*z*+2; (iv) −*x*+1, *y*+1/2, −*z*+3/2; (v) *x*, −*y*+3/2, *z*+1/2.
