# Supplementary figures and images for: Crystal structure of bis­[2-(1H-benzimid­azol-2-yl)-4-bromo­phenolato-κ2 N 3,O]cobalt(II)
Source: Acta Crystallogr Sect E Struct Rep Online. 2014 Oct 11;70(Pt 11):m363–4. doi: 10.1107/S1600536814021813 (PMC4257236; doi:10.1107/S1600536814021813)

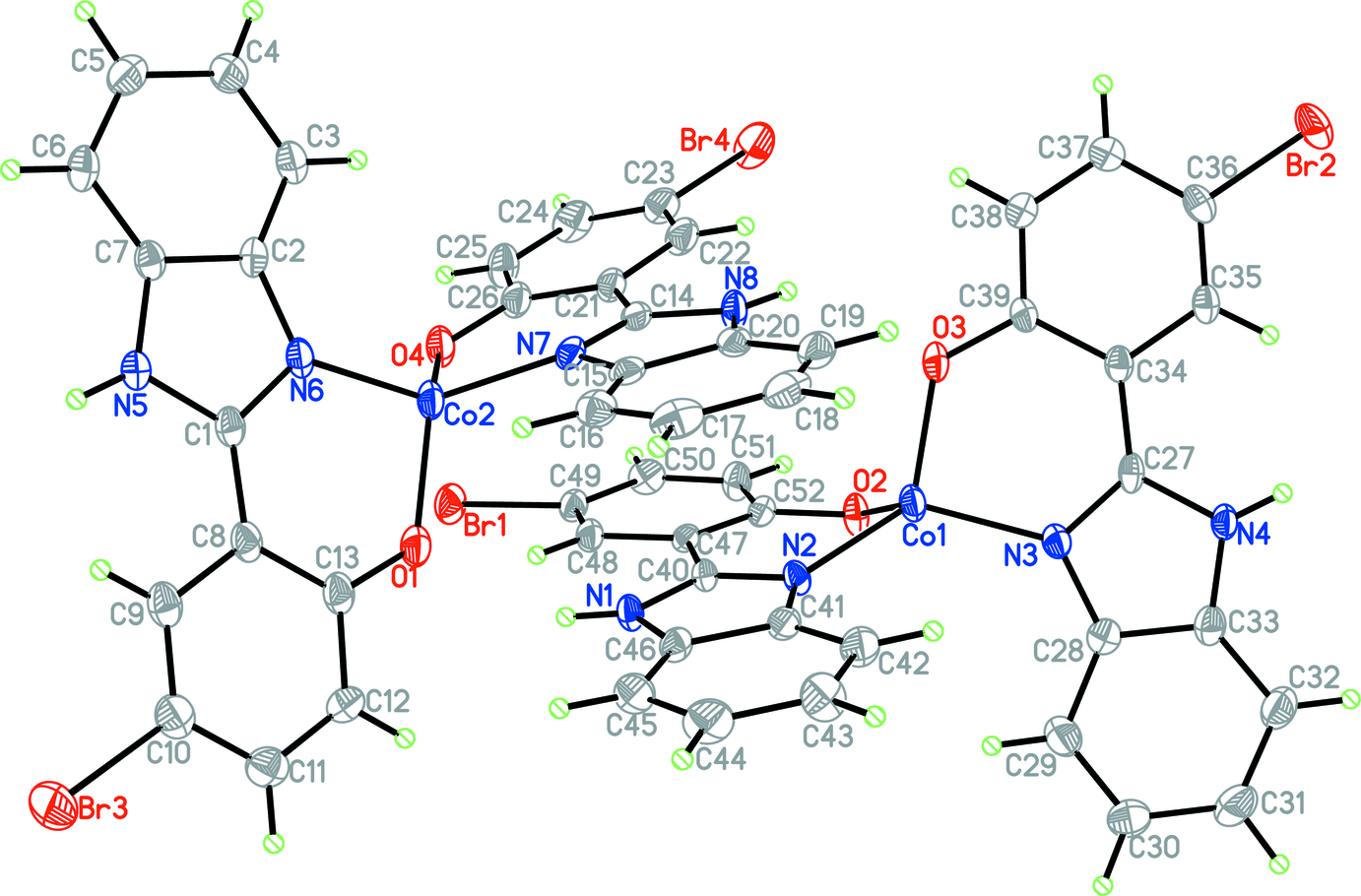

Supplement: Supplementary file 3 [file e-70-0m363-fig1.tif]

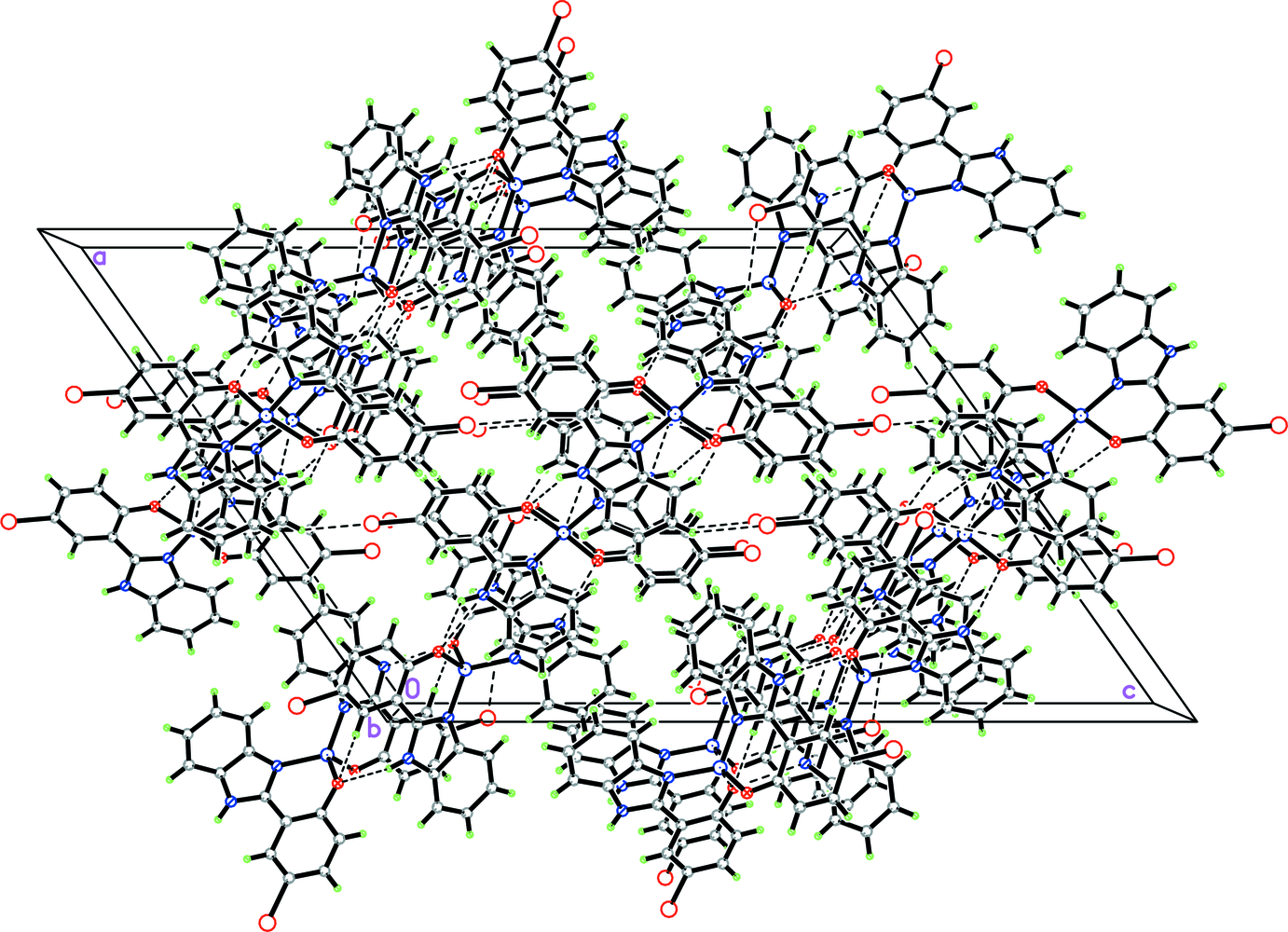

Supplement: Supplementary file 4 [file e-70-0m363-fig2.tif]
